# Therapeutic efficacy of a MMAE-based anti-DR5 drug conjugate Oba01 in preclinical models of pancreatic cancer

**DOI:** 10.1038/s41419-023-05820-1

**Published:** 2023-04-29

**Authors:** Chao Zheng, Dongdong Zhou, Weisong Li, Yanhui Duan, Minwen Xu, Jie Liu, Jingpei Cheng, Youban Xiao, Han Xiao, Tao Gan, Jianmin Liang, Dexian Zheng, Liefeng Wang, Shuyong Zhang

**Affiliations:** 1 Key Laboratory of Prevention and Treatment of Cardiovascular and Cerebrovascular Diseases, Ministry of Education, Gannan Medical University, Ganzhou, 341000 China; 2 School of Basic Medicine, Gannan Medical University, Ganzhou, 341000 China; 3 Department of Pathology, Clinical Skill Center, First Affiliated Hospital, Gannan Medical University, Ganzhou, 341000 China; 4 Yantai Obioadc Biomedical Technology Ltd., Yantai, 264000 China

**Keywords:** Targeted therapies, Preclinical research

## Abstract

Pancreatic cancer (PC) is among the most aggressive malignancies associated with a 5-year survival rate of <9%, and the treatment options remain limited. Antibody–drug conjugates (ADCs) are a new class of anticancer agents with superior efficacy and safety profiles. We studied the antitumor activity of Oba01 ADC and the mechanism underlying the targeting of death receptor 5 (DR5) in preclinical PC models. Our data revealed that DR5 was highly expressed on the plasma membrane of PC cells and Oba01 showed potent in vitro antitumor activity in a panel of human DR5-positive PC cell lines. DR5 was readily cleaved by lysosomal proteases after receptor-mediated internalization. Monomethyl auristatin E (MMAE) was then released into the cytosol to induce G2/M-phase growth arrest, cell death via apoptosis induction, and the bystander effect. Furthermore, Oba01 mediated cell death via antibody-dependent cell-mediated cytotoxicity and complement-dependent cytotoxicity. For improved potency, we investigated the synergetic effect of Oba01 in combination with approved drugs. Oba01 combined with gemcitabine showed better antiproliferative activity than either standalone treatment. In cell- and patient-derived xenografts, Oba01 showed excellent tumoricidal activity in mono- or combinational therapy. Thus, Oba01 may provide a novel biotherapeutic approach and a scientific basis for clinical trials in DR5-expressing patients with PC.

## Introduction

Pancreatic cancer (PC) is a highly malignant tumor known as the “king of cancer” [1]. PC does not have a high incidence rate; however, the degree of malignancy is very high, especially because the symptoms are not obvious and it occurs in the deepest part of the abdomen. On the appearance of the symptoms, ~60% of patients enter into the advanced PC stage [2]. Tumors of >90% of patients are unresectable at this stage, and the 5-year survival rate is <9% [3]. Chemotherapy remains the most important current treatment option for PC, but its therapeutic effects are limited [4–6]. Therefore, new therapeutic technologies, drug targets, and the development of effective targeting drugs are urgently required.

Therapeutic monoclonal antibodies have led to breakthroughs in treating solid tumors and hematologic malignancies. In particular, antibody–drug conjugates (ADCs), also known as “bio-missiles,” are monoclonal antibodies coupled with a cytotoxic agent via a plasma-stable linker. ADCs can bind to the tumor-associated antigen on the cell surface and are subsequently endocytosed into lysosomes to release the toxin payload by cleaving the protease-mediated linker to inhibit tumor cells [7, 8]. A total of 14 ADC drugs have been globally approved to date, including Mylotarg, Adcetris, Kadcyla, Besponsa, Lumoxiti, Polivy, Padcev, Enhertu, Trodelvy, Blenrep, Akalux, Lonca, RC48, and Tivdak [9, 10], which are used to treat several tumor types, such as leukemia, lymphoma, multiple myeloma, breast cancer, head and neck cancer, cervical carcinoma, gastric cancer, and urothelial carcinoma [11, 12].

Tumor necrosis factor-related apoptosis-inducing ligand receptor 2, also known as death receptor 5 (DR5), is a type I membrane molecule of the tumor necrosis factor receptor superfamily [13]. DR5 shows low or no expression in normal cells; however, it is highly expressed in various cancers, including lymphocytic leukemia, lymphoma, multiple myeloma, lung carcinoma, colon cancer, breast cancer, ovarian cancer, bladder cancer, and pancreatic cancer [14, 15]. DR5 can particularly induce apoptotic and autophagic cell death in various tumor cells via an extrinsic or intrinsic signaling pathway [16–18]. Despite progress in the clinical development of agonistic anti-DR5 monoclonal antibodies, these antibodies have not reached the market [19, 20].

We have previously reported a novel potent ADC based on an anti-DR5 antibody (zaptuzumab), zaptuzumab-PY-VC-MMAD, named Zapadcine-1. Zapadcine-1 exhibited excellent antitumor activity against acute lymphoblastic leukemia (ALL); however, its safety window was relatively small because the MMAD payload was more toxic than other members of the auristatin cancer drug family [21]. On structural optimization and screening, we discovered a new candidate ADC, zaptuzumab-PY-VC-MMAE, named Oba01. Our results reveal that Oba01 retains the tumor specificity of zaptuzumab and can be localized in lysosomes via endocytosis. Similar to Zapadcine-1, Oba01 significantly inhibits tumor growth in DR5-positive lymphocytic leukemia in vitro and in vivo in a dose-dependent manner. Its safety window, when compared with Zapadcine-1, was significantly larger in both acute and chronic toxicity studies in rats and cynomolgus monkeys, suggesting that Oba01 has excellent preclinical tolerability and a good safety profile [22]. Therefore, it can be considered an optimistic prospect for subsequent clinical development.

We have reported that Oba01 demonstrates encouraging preclinical antitumor activity in DR5-positive ALL; however, its potential applications in solid tumor treatment have not been well investigated. Here, we present the first report of preclinical data supporting Oba01 as a novel therapeutic candidate for PC. We first quantified the expression of DR5 in PC cell lines, patient tissues, and normal tissues; subsequently, we demonstrated the antitumor activities of Oba01 and their underlying mechanisms in PC. The results revealed that Oba01 has excellent antitumor activity in various PC cell lines, cell-derived xenografts (CDXs), and patient-derived xenografts (PDXs). Oba01 combined with gemcitabine had synergistic effects in vitro and in vivo. Our study provides strong evidence for the clinical advancement of Oba01 as a novel biotherapeutic for DR5-expressing patients with PC.

## Materials and methods

### Drugs and reagents

Oba01, Zaptuzumab (naked antibody of Oba01), and MMAE were generated by Yantai Mabplex International Bio-Pharmaceutical Co. Ltd. (Shandong, China). Gemcitabine was purchased from Selleck (Houston, USA). Primary antibodies are: anti-LAMP1(Thermo Fisher Scientific, Rockford, USA); anti-mTOR (Proteintech, 28273-1-AP), anti-Phospho-mTOR (Ser2448) (CST, 2971), anti-AKT (CST, 9272), anti-Phospho-Akt (Ser473) (D9E) (CST, 4060), anti-PARP (46D11) (CST, 9532), anti-Mcl-1 (D2W9E) (CST, 94296), anti-DR5 (E9D7D) (CST, 69400), anti-Bcl-2 (Affinity, AF6139), anti-Rb (Affinity, AF6103), anti-Phospho-Rb (Affinity, AF3103), anti- Cyclin E1 (Affinity, AF0144), anti- CDK4 (Proteintech, 11026-1-AP), anti-Cyclin D1 (Proteintech, 26939-1-AP), anti-GAPDH (CST, 5174); anti-DR5 mAb (TA807279, OriGene Technologies, Inc). Secondary antibodies are: HRP-labeled donkey anti-human IgG-H&L, and goat anti-rabbit HRP conjugated IgG-H& L (Abcam, ab6721).

### Cell lines

The human pancreatic cancer cells Mia PaCa-2, PL45, PATU8988, JF305, PANC-1, Panc 10.05, Panc 05.04, BxPC-3, the human bladder cancer cell T24, and the human lung cancer cell Calu-1 were purchased from iCell Bioscience Inc. (Shanghai, China) or the Cell Bank of Chinese Academy of Medical Sciences (Beijing, China). All the cell identity was validated by short tandem repeats (STR) and confirmed to be free of mycoplasma contamination. All the cells were cultured in RPMI-1640 medium (Gibco) supplemented with 10% fetal bovine serum (Gibco) and 1% penicillin/streptomycin (Solarbio, Shanghai, China) in a humidified incubator (Thermo Fisher Scientific, Waltham, MA) with 5% CO_2_ at 37 °C.

### RNA isolation and real-time quantitative PCR analysis

Total RNA was isolated with RNAiso plus reagent (TaKaRa, Japan). Real-time quantitative PCR was performed by using the TB Green® Premix Ex Taq™II (Takara, Japan), PrimeScript™RT reagent kit with gDNA Eraser (Takara) was used for reverse transcription and the relative quantification of mRNA. Specific primers were listed as follows, Human DR5, Forward primer: 5′-AAGTGGAGCTAAGTCCCTGC-3′, Reverse primer: 5′-CCCACTGTGCTTTGTACCTG-3′. Human β-actin, Forward primer: 5′-GCCAACACAGTGCTGTCTGG-3′, Reverse primer: 5′-CTCAGGAGGAGCAATGATCTTG-3′. The data were collected and analyzed by using the QuantStudio 5 Apparatus (ABI). β-actin was used as a control, and the relative expression was calculated by using the comparative Ct method, while the relative expressions were analyzed by the 2^−△△Ct^ method.

### Western blot

Cells were lysed by SDS lysis buffer (Beyotime Biotechnology, Shanghai, China) supplemented with PMSF (Solarbio, Beijing, China) and Phosphatase Inhibitor Cocktail (Beyotime Biotechnology, Shanghai, China). Proteins were diluted in loading buffer (Solarbio, Beijing, China) and isolated by SDS-PAGE, then transferred onto PVDF membranes (Millipore, Darmstadt, Germany). The membranes were incubated with the primary antibody overnight at 4 °C and then incubated with secondary antibodies specific to the primary antibody. The protein band was detected by an ECL reagent (Cytiva, USA). Images were taken using a Bio-Rad Multifunctional chemiluminescence imaging system.

### In vitro cytotoxicity assay

A panel of PADC cell lines was treated with a tenfold dilution series of Oba01 in triplicate in media supplemented with 10% heat-inactivated bovine serum (Gibco) for 3 days. The cell viability was measured with the cell Titer-Glo® assay kit (G7572, Promega, Madison, USA) in accordance with the manufacturer’s instruction, while the absorbance was determined by the SPARK Multiplate Reader (TECAN, Switzerland). The untreated cells served as a control. The cell survival rate (%) was calculated using the following formula: Asample/A control × 100%. The 50% inhibitory concentration (IC50) was calculated by nonlinear regression analysis (GraphPad Prism 5.0). The coefficient of drug interaction (CDI) was calculated to evaluate the interaction between two drugs, as reported previously [23].

### Internalization assay

Internalization assay was performed using a protocol as described previously [22].

### Cell apoptosis and cycle arrest assay

For the apoptosis assay, Mia PaCa-2 and PL45 cells were seeded at the density of 2 × 10^5^ cells/well and exposed to Oba01 at 0, 0.2, 1, and 5 μg/mL for 48 h, and the cell death was detected by using the Annexin V-FITC Apoptosis Kit (Beyotime Biotechnology, Shanghai, China). For the cell cycle analysis, Mia PaCa-2 and PL45 cells were seeded at the density of 2 × 10^5^ cells/well and exposed to Oba01 at 0, 1, 5, and 25 μg/mL for 48 h, and the total DNA content was detected by using the cell cycle assay kit (Beyotime Biotechnology). The cell cycle position and apoptosis analyses were measured by flow cytometry (BD, FACSCanto II, USA).

### Clonogenic assay

Mia PaCa-2 WT/knockout and T24 WT/overexpression cells were seeded in a six-well plate at the density of 3000 cells/well and treated with 0, 1, and 5 μg/mL Oba01. The six-well plate was incubated at 37 °C under a 5% CO_2_ atmosphere for 1 week. Then, the colonies were rinsed with PBS, fixed with 4% paraformaldehyde for 10 min at room temperature, and stained with 0.01% (w/v) crystal violet for 20 min at room temperature. The number of colonies containing at least 50 cells was enumerated.

### ADCC and CDC assay

For the ADCC assay, Mia PaCa-2 and PL45 cells were seeded in a 96-well plate at the density of 5 × 10^3^ cells/well with DMEM supplemented with 10% FBS and then treated with Oba01 for 30 min. Next, the cells were incubated with human peripheral blood mononuclear cells (PBMCs) (target cells: effector cells ratio 1:5, 1:20, 1:80) for 4 h. For the CDC assay, the serum samples were first separated from the whole blood samples obtained from two healthy volunteers. A half portion of each sample was incubated in a 56 °C water bath for 30 min to inactivate the complement activity. Mia PaCa-2 and PL45 cells were seeded in a 96-well plate at the density of 5 × 10^3^ cells/well with the DMEM culture medium supplemented with 10% FBS and then treated with Oba01 for 30 min. Subsequently, the cells were incubated with the prepared human serum samples (final concentration 20%) for 4 h. After incubation, the cells were lysed with lysis buffer for 45 min, and the CytoTox 96^®^ Non-Radio Reagent (Promega) was added to measure the OD_490_ values on the SPARK Multiplate Reader (TECAN, Switzerland), after which the lysis rate (%) was calculated.

### Bystander killing

Two cell lines were generated using a lentiviral vector expression system: the DR5-positive Mia PaCa-2 and PL45 cell lines stably expressed the green fluorescent protein (GFP) and the Calu-1 cell line with less or no DR5 expression stably expressed the red fluorescent protein (mCherry). These cell lines were independently confirmed for in vitro sensitivity to Oba01 and demonstrated that Oba01 could kill the Mia PaCa-2/GFP and PL45/GFP cells, but not the Calu-1/mCherry cells. Next, Mia PaCa-2/GFP and Calu-1/mCherry or PL45/GFP and Calu-1/mCherry cells were cultured alone and mixed at a 2:1 ratio overnight. The cells were then treated with Oba01 for 5 days and live cells were detected by flow cytometry (BD, FACSCanto II, USA).

### DR5 knockdown and overexpression

To establish a stable DR5 knock-out Mia PaCa-2 cell line, *DR5* was knocked down by CRISPR/Cas9 gene editing mediated via electrotransfection using gRAN1, gRNA2, and gRNA1/gRNA2. Then, the homozygous Mia PaCa-2 cells with DR5 knockdown were successfully obtained through monoclonal selection and verified by sequencing, qPCR, and Western blotting. The gRNA1 target sequence was: 5′-ATAGTCCTGTCCATATTTGCAGG-3′ and the gRNA2 was: 5′-CGGCACTTCCGGCACATCTCAGG-3′. To establish a stable T24 DR5 overexpression cell line, the cells were transduced by using a lentiviral vector expression system with the DR5 cDNA sequence. Stable DR5 overexpression T24 cells were selected with puromycin (2 μg/mL) for at least 7 days and verified by qPCR and Western blotting.

### Animal studies

Female BALB/c nude mice (age: 5–6 weeks) were purchased from SJA Laboratory Animal Co., Ltd. (Hunan, China) and housed and maintained under SPF conditions. All animal experiments were approved and performed in complete compliance with the guidelines approved by the Biomedical Research Ethics Committee, Gannan Medical University (Jiangxi, China) or Crown Bioscience Inc. (Beijing, China). All mice were fed with standard laboratory chow and provided water *ad libitum*. For the CDX models, 5 × 10^6^ PC cells, including Mia PaCa-2, PL45, PATU8988, and JF305, respectively, were suspended in 100 μL of PBS and injected subcutaneously into the right flank of the experimental mice. For the PDX models, the mice were inoculated subcutaneously in the right flank with tumor fragments (2–3-mm diameter) by Crown Bioscience Inc. (Beijing, China). When the mean tumor volume reached 100–150 mm^3^, the mice were randomized to 3–5 groups (*n* = 4–6/group) and injected intravenously with Oba01 at different doses with QW × 3 manner. Then, the mice intravenously received saline, Oba01 (5.0 and 10 mg/kg), zaptuzumab (10 mg/kg), and MMAE (0.2 mg/kg, equimolar MMAE in the Oba01 10 mg/kg dose). In addition, for the combination therapy, BALB/c nude mice were treated with vehicle, 5 mg/kg Oba01, 25 or 40 mg/kg gemcitabine, and their combination, at the same doses as mentioned above. The tumor sizes and body weight were measured twice a week with a caliper, and the tumor volumes were determined according to the following formula: tumor volume (mm^3^) = length × (width)^2^ × 0.5. T/C was calculated as the treated tumor volume/control tumor volume × 100%. The inhibition rate of tumor growth (TGI) was calculated as (1 − T/C) × 100%.

### Histopathology and immunohistochemistry analysis

PDAC patient tissues were prepared into 4-μm-thick paraffin tissue microarrays (TMA) (US Biomax) by using Human on human IHC Kit and DAB Substrate Kit (Abcam, Cambridge, USA) and subjected to hematoxylin and eosin (H&E) staining as per the standard procedure. Imaging was performed under a TissueFAXS Plus (version 7.0, TissueGnostics GmbH, Vienna, Austria).

### Statistical analysis

All experimental data were presented as the mean ± standard error of mean (SEM) of three independent experiments by using GraphPad Prism 5 software. IC50 values were determined through nonlinear regression analysis of concentration-response curves using SPSS 16.0. The statistical significance between the two groups was analyzed by one-way analysis of variance (ANOVA) or Student’s *t*-test when compared with the untreated or vehicle-control group. For all experiments, differences were considered to be significant at *P* < 0.05. *0.01 ≤ *P* < 0.05; **0.001 ≤ *P* < 0.01; ***P < 0.001.

## Results

### Overexpression of DR5 in PC cells

**Fig. 1:**
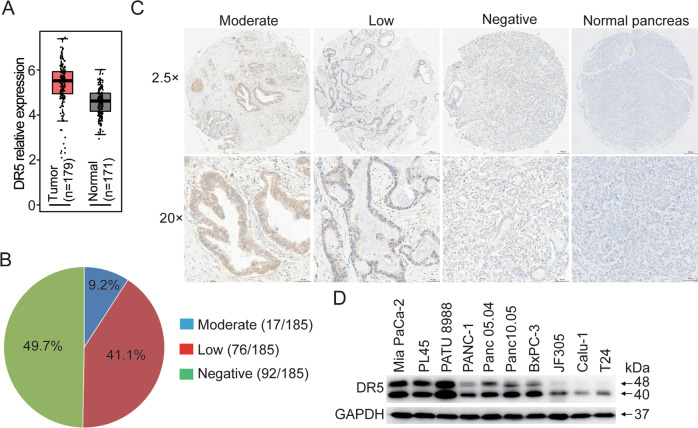
DR5 is overexpressed in pancreatic cancer. **A** DR5 mRNA overexpression in human PC versus normal pancreas. The box plot has been downloaded from the GEPIA database. **B** Percent of 185 PADC tumors in each of the three staining intensity, including moderate, low, and negative. **C** Representative pictures of the three staining intensity and one normal pancreas of DR5 IHC on human PADC tumor tissue microarray. **D** The DR5 protein expression in a panel of PC cell lines and other solid tumor cells assessed by Western blotting. Images were captured at 25× magnification (upper panels) and 200× magnification (lower panels). Scale bars = 200 µm (upper panels). Scale bars = 50 µm (lower panels).

We first sought to characterize the expression of DR5 in PC as a potential ADC target using the GEPIA database [24]. DR5 expression was significantly upregulated in PC tissues compared with normal pancreatic tissues (Fig. 1A). DR5 has reportedly shown overexpression in pancreatic ductal carcinoma (PDAC) [25–27]. We performed IHC analyses of DR5 from 185 human PDAC patient specimens using 3 tissue microarrays (TMAs) (Fig. 1B). DR5 was positive in 50.3% (93/185) of TMA PDAC patient specimens. In particular, DR5 showed moderate, low, and negative staining intensities in 9.2% (17/185), 41.1% (76/185), and 49.7% (92/185) of specimens, respectively. IHC staining revealed considerable variability in DR5 expression in tumor cells from PDAC specimens, whereas DR5 was not detected in five normal human pancreatic tissues. Figure 1C showed IHC staining images of PDAC TMAs, which showed moderate, low, or negative staining. We also found various subcellular staining spots of DR5 in PDAC cells. DR5 was found to be predominantly localized on the cell membrane and partially retained within the cytoplasm of PDAC tumor cells. Furthermore, all CDXs (Figs. 4E, J, 6D, G) and PDXs (Fig. 7D, G) tested in standalone and combination therapies showed positive DR5 expressions that were predominantly membrane-localized. Altogether, these results confirm that DR5 was widely expressed on the cell surfaces of PDAC CDX/PDX tumors, and its expression appeared to be restricted to tumor tissues.

We screened for relevant PC cell lines for in vitro and in vivo evaluation. DR5 protein expressions were determined in a panel of PC and non-PC solid tumor cell lines using western blotting. DR5 was found to be highly expressed in Mia PaCa-2, PL45, PATU8988, PANC-1, JF305, BxPc-3, Panc 05.04, and Panc 10.05 cell lines, but not in Calu-1 and T24 cell lines (Fig. 1D).

### DR5-mediated antitumor activity of Oba01 in PC cells in vitro

The ability of being internalized is one of the key requirements for the drugability of ADC drugs. Our endocytosis assays revealed that Oba01 induced DR5 internalization in Mia PaCa-2 and PL45 cells (Supplementary Fig. 1). We evaluated the cytotoxicity induced by Oba01 in a panel of human PC cells after internalization (Supplementary Table 1 and Supplementary Fig. 2). Oba01 was highly cytotoxic with IC_50_ values in the range of 4.79 ± 1.43 to 347.55 ± 150.54 nM in Mia PaCa-2, PL45, PATU8988, PANC-1, JF305, BxPc-3, Panc 05.04, and Panc 10.05 cells. However, we observed no inhibitory effects on T24 and Calu-1 cells. Furthermore, the payload MMAE led to significant inhibition of all tested cells with IC_50_ values in the range of 0.06 ± 0.01 to 0.89 ± 0.24 nM, irrespective of the level of DR5 expression. Therefore, Oba01 showed good cytotoxicity against DR5-positive PC cells.

**Fig. 2:**
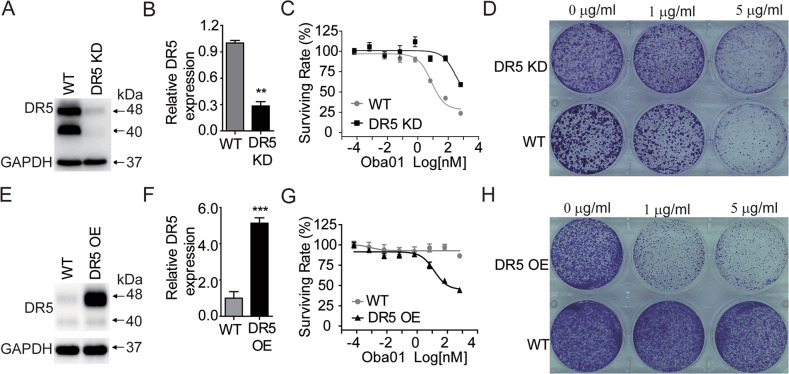
Effects of DR5 knockdown (KD) and overexpression (OE) in PC cells on sensitivity to Oba01. **A**, **B** Protein and mRNA expression of DR5 in Mia PaCa-2 control and CRISPRi DR5 KD Mia PaCa-2 cells. **C** Cytotoxicity assay of Oba01 on Mia PaCa-2 control and DR5 KD cells. **D** Representative images of the clonogenicity assay in Mia PaCa-2 control and DR5 KD cells treated with Oba01. **E**, **F** DR5 protein and mRNA expression in T24 control and DR5 OE cells. **G** Cytotoxicity assay of Oba01 on T24 control and DR5 OE cells. **H** Representative images of the clonogenicity assay in T24 control and DR5 OE cells treated with Oba01.

To investigate whether the cytotoxicity of Oba01 is DR5-dependent, we generated DR5-knockdown Mia PaCa-2 cells by the CRISPR/Cas9 system using three unique DR5-specific sgRNAs. All three sgRNAs efficiently silenced DR5 protein expression, indicating Oba01 insensitivity (Supplementary Fig. 3). We subsequently obtained the subclones of DR5 gRNA1 in Mia PaCa-2 and PL45 cells via screening. Western blotting and qPCR confirmed that gRNA1 efficiently silenced DR5 mRNA and protein expression (Fig. 2A, B and Supplementary Fig. 4A, B). DR5-silenced Mia PaCa-2 and PL45 cells were resistant to Oba01 compared to control cells with significant DR5 expression (Fig. 2C and Supplementary Fig. 4C).

We next evaluated whether DR5 overexpression could enhance Oba01-induced cell death. DR5 was ectopically expressed using the lentivirus-mediated method in the T24 and Calu-1 cell lines (Fig. 2E, F and Supplementary Fig. 4D, E). The upregulation of DR5 expression in T24 and Calu-1 cell lines led to a marked increase in Oba01-mediated cytotoxicity (Fig. 2G and Supplementary Fig. 4F). Furthermore, clonogenic assays confirmed that the cytotoxicity mediated by Oba01 was DR5-dependent (Fig. 2D, H).

Thus, our data demonstrated that the intrinsic expression of DR5 played a crucial role in determining cellular sensitivity toward Oba01.

### Oba01 exerts in vitro antitumor activity by inducing apoptosis, the bystander effect, ADCC, and CDC

**Fig. 3:**
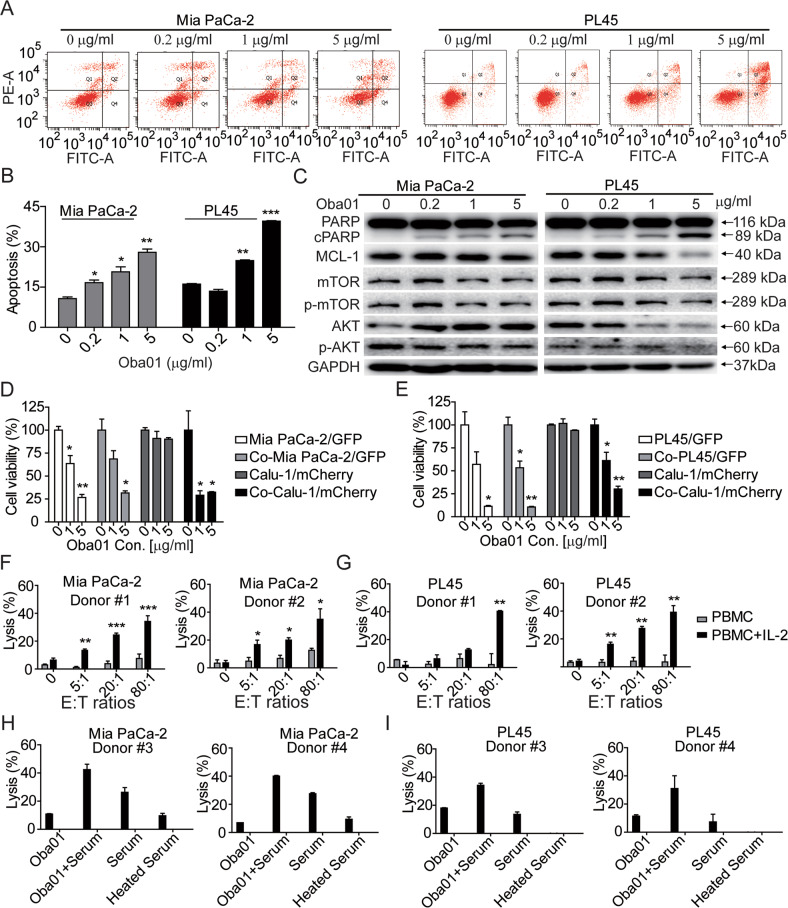
Oba01 induced cell death in pancreatic cancer cells. **A**, **B** Mia PaCa-2 and PL45 cells were treated with different concentrations of Oba01 (as indicated) for 48 h. Cell apoptosis was assessed by flow cytometry and percent of Annexin V/PI-positive cells are depicted in bar charts. **C** Cell lysates were subjected to immunoblotting with antibodies to p-AKT, AKT, P-mTOR, mTOR, MCL-1, PARP, or with antibodies to GAPDH as the loading control. **D**, **E** Oba01 demonstrated a strong bystander killing activity. Mia PaCa-2/GFP cells (**D**) or PL45/GFP cells (**E**) and Calu-1/mCherry cells were cultured alone or mixed at a ratio of 2.5:1 and cocultured (Co) overnight, followed by treatment with various concentrations of Oba01 for 5 days. Live cells was detected by flow cytometry. **F**, **G** Oba01 mediated the ADCC activity. Mia PaCa-2 (**F**) and PL45 cells (**G**) were plated in a 96-well plate, and IL-2-activated human PBMCs were added at different E:T ratios (E: PBMC, T: Mia PaCa-2 or PL45 cells) to detect the ADCC. **H**, **I** Oba01 mediated the CDC activity. Human serum was added to Mia PaCa-2 (**H**) and PL45 cells (**I**) in the presence or absence of Oba01. Heat-inactivated serum was used as a negative control.

To explore the mechanisms underlying the antitumor activity of Oba01 in vitro, we analyzed apoptosis, cell cycle arrest, bystander killing, ADCC, and CDC activities. First, we observed dose-dependent apoptotic cell death in DR5-positive Mia PaCa-2 and PL45 cells (Fig. 3A, B). The level of PARP cleavage increased consistently while the levels of MCL-1, p-mTOR, and p-AKT decreased on Oba01 treatment (Fig. 3C). Furthermore, Oba01 induced changes in the cell cycle characterized by a decreased G0–G1 phase and an increased G2–M-phase in DR5-positive Mia PaCa-2 and PL45 cells (Supplementary Fig. 5).

ADC-mediated bystander killing is facilitated by the free cytotoxins that passively diffuse from the target-positive cancer cells into the tumor microenvironment, killing adjacent cancer cells [28]. To evaluate the bystander effect of Oba01 in vitro, we cocultured DR5-positive, Oba01-sensitive Mia PaCa-2/GFP cells or PL45/GFP cells along with DR5-negative, Oba01-resistant T24/mCherry cells. Both DR5-positive and DR5-negative cells were efficiently inhibited by Oba01, confirming that Oba01 has bystander effects (Fig. 3D, E).

To further understand the mechanisms underlying Oba01, we evaluated ADCC and CDC activities. We observed Oba01-mediated ADCC in Mia PaCa-2 and PL45 cells with an increase in the E:T (E: PBMCs, T: PC cells) ratio (Fig. 3F, G). We simultaneously conducted a CDC assay using human serum as a compliment. The serum alone induced lysis of 26.29 ± 3.39% to 27.54 ± 0.81% and 7.66 ± 5.47% to 13.57 ± 1.73%, whereas in the presence of Oba01, we observed lysis of 40.01 ± 0.55% to 42.39 ± 3.88% and 34.16 ± 1.45% to 31.02 ± 9.19% in Mia PaCa-2 and PL45 cells, respectively. However, on using heat-inactivated serum as a negative control, 9.44 ± 1.63% to 9.64 ± 1.69% or 0% lysis was observed (Fig. 3H, I). Therefore, ADCC and CDC were involved in the antitumor activity of Oba01.

Altogether, our results showed that Oba01 inhibited cancer cells via a combined mechanism of apoptosis, cell cycle arrest, bystander effect, and ADCC and CDC activities.

### Standalone antitumor activity of Oba01 in PC CDX models

**Fig. 4:**
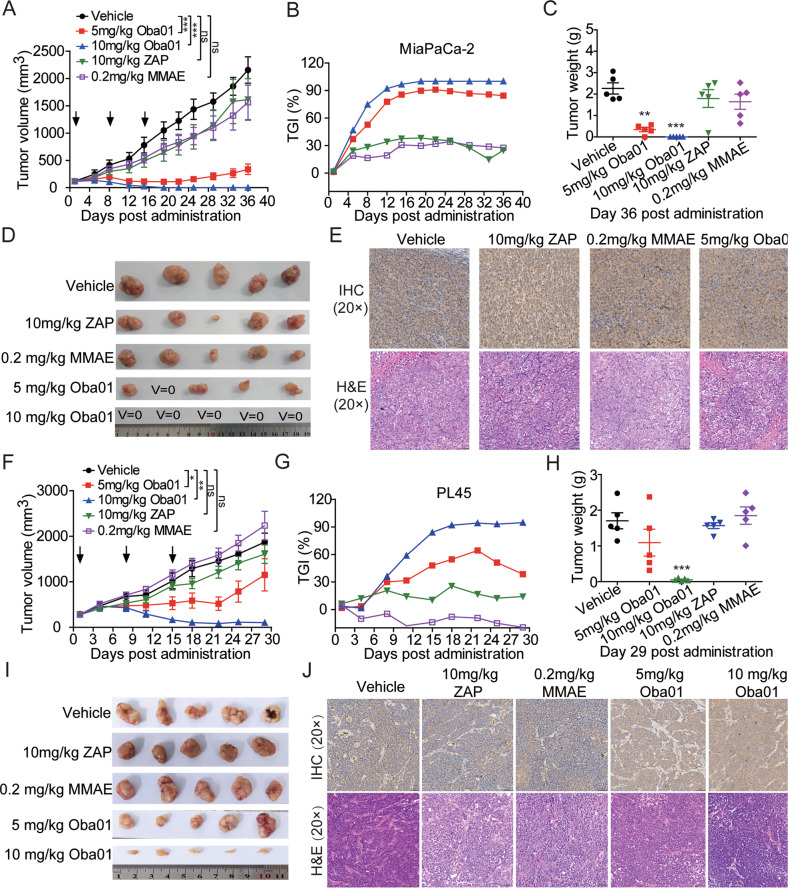
Antitumor activity of Oba01 in mouse CDX models. **A**, **B** Tumor growth curve and inhibition rate of tumor growth (TGI) in a Mia PaCa-2 CDX model. **C**, **D** Transplanted tumor weights and tumor images were evaluated at the end of the experiment in the Mia PaCa-2 CDX model. **E** IHC analysis of DR5 expression (upper panels) and H&E staining (lower panels) in the transplanted tumors derived from the mice shown in panel 4D. **F**, **G** Tumor growth curve and TGI in the PL45 CDX model. **H**, **I** Transplanted tumor weights and tumor images assessed at the end of the experiment in the PL45 CDX model. **J** IHC analysis of DR5 expression (upper panels) and H&E staining (lower panels) in the transplanted tumors derived from the mice shown in panel 4I. IHC and H&E Images were captured at 200×magnification, respectively. Scale bars = 50 µm.

We performed a set of in vivo efficacy studies to evaluate the therapeutic potential of Oba01. Oba01 administration induced significant tumor regression in Mia PaCa-2 and PL45 CDX models. Terminal tumor growth inhibition (TGI) of 84.37% and 51.13% was observed at a dose of 5 mg/kg in Mia PaCa-2 and PL45 CDX models, respectively. Complete regression (CR) was observed in five of five animals in the groups administered 10 mg/kg Oba01 on day 12 in the Mia PaCa-2 model (Fig. 4A–D), and 10 mg/kg Oba01 resulted in a TGI of 92.94% in the PL45 model (Fig. 4F–I). In contrast, the payload MMAE (0.2 mg/kg, equimolar MMAE in the 10 mg/kg dose of Oba01) and zaptuzumab at 10 mg/kg had no inhibitory effects on tumor growth. Furthermore, mouse body weight showed no significant changes with any of the treatments (Supplementary Fig. 6). All CDX tumors were collected and prepared for IHC and H&E assays. DR5 was expressed in all CDX tumors, indicating that the superior antitumor activity of Oba01 was DR5-dependent (Fig. 4E, J). Histopathological images of all mouse organ dissections showed that Oba01 had no toxic adverse effects on the heart, liver, spleen, lung, and kidney (Supplementary Fig. 7). Similarly, the superior antitumor efficacy of Oba01 was also validated in two other subcutaneous xenograft models of PATU8988 and JF305 (Supplementary Figs. 8, 9). Therefore, these results established that Oba01 shows excellent in vivo efficacy in a DR5-dependent manner in PC CDX models.

### Gemcitabine sensitizes Oba01-mediated cytotoxicity

**Fig. 5:**
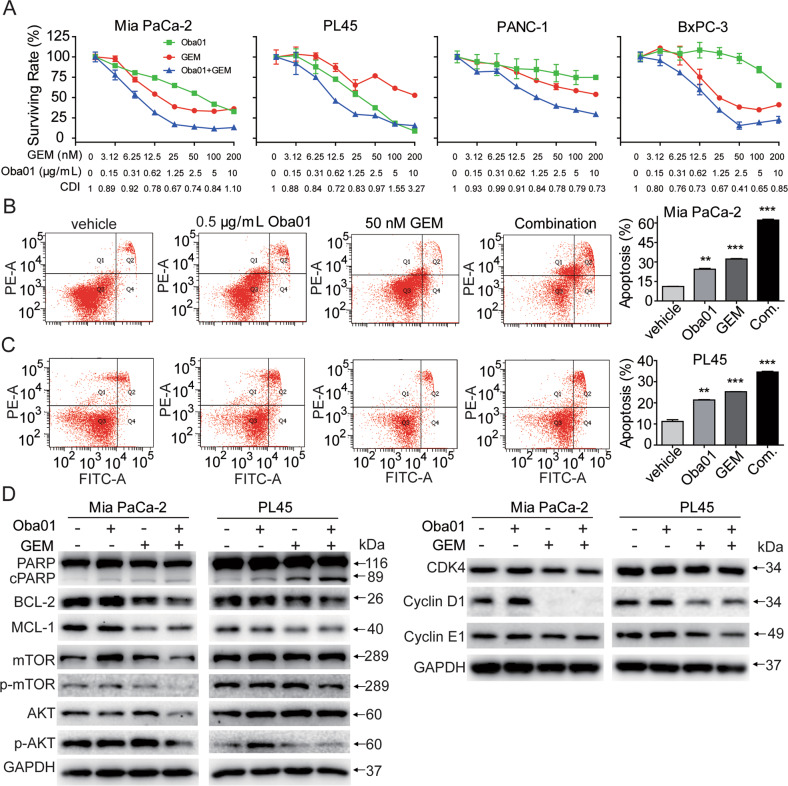
Cell apoptosis induced by Oba01 and/or gemcitabine in DR5-positive pancreatic cancer cells. **A** Cytotoxicity of a combination with Oba01 and gemcitabine (GEM) in a panel of PC cell lines as determined by CellTiter-Glo® Assay in accordance with the manufacturer’s instructions. All results are presented as the mean of three independent experiments ± SD. CDI <1 represents a synergistic effect. **B**, **C** Mia PaCa-2 and PL45 cells were treated with Oba01, GEM, or their combination (Com.) for 48 h. The cells were stained with an anti-Annexin V-FITC antibody and PI for apoptosis analysis by flow cytometry. Annexin V-FITC-positive cells were defined as apoptotic. **D** Mia PaCa-2 and PL45 cells were treated with the indicated concentrations of Oba01 and GEM or their combination for 48 h. The cell lysates were subjected to immunoblotting analysis with antibodies to p-AKT, AKT, P-mTOR, mTOR, BCL-2, MCL-1, PARP, CDK4, CDK4, Cyclin D1, Cyclin E1, or with antibodies to GAPDH as the loading control.

To improve the potency of Oba01, we combined Oba01 with a panel of FDA-approved drugs in preclinical or clinical trials. Several combinations showed synergistic effects in Mia PaCa-2 and PL45 cells (Supplementary Table 2). Among them, gemcitabine showed the most superior synergetic effect with Oba01 in a panel of PC cell lines (Fig. 5A–C). To further explore the mechanisms underlying this synergy, we examined markers for apoptosis, cell cycle, and the activation of the PI3K/AKT/mTOR pathway using immunoblotting techniques. Similar to Oba01-treated cells, cleaved PARP levels increased with the combined treatment of the two drugs in both Mia PaCa-2 and PL45 cells. This synergistic effect was accompanied by significantly decreased phosphorylation of AKT and mTOR along with the downregulated expressions of CDK4, cyclin D1, and cyclin E1, consistent with MMAE and gemcitabine-induced G2/M cycle blocking (Fig. 5D). Therefore, the enhanced cytotoxicity observed with the Oba01/gemcitabine combination mechanistically involved BCL-2 induction, MCL-1 and PARP-dependent apoptosis, CDK4, cyclin D1, and cyclin E1-dependent cell cycle arrest, and suppression of the PI3K/mTOR pathway.

### Oba01 and gemcitabine demonstrated a synergistic effect in CDXs and PDXs

**Fig. 6:**
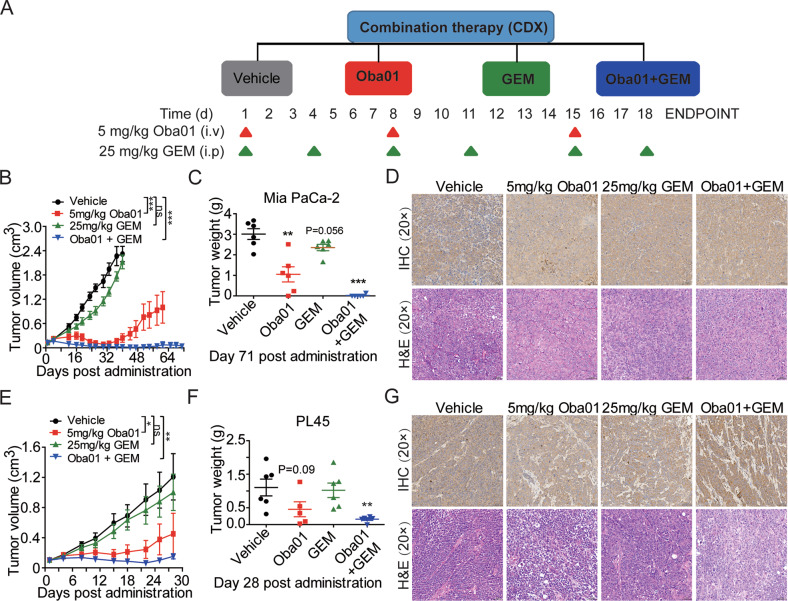
Combination of Oba01 and gemcitabine significantly inhibited pancreatic cancer tumor growth in Mia PaCa-2 and PL45 CDXs. **A** Schematic representation of the experimental designs. Treatment groups, doses, and schedules are shown. Oba01 (red line; 5 mg/kg) and vehicle (black line) injections were administered intravenously and gemcitabine (GEM, green line) was administered intraperitoneally; the combination group is depicted as a blue line. **B**, **C** Tumor growth curve and the transplanted tumor weight was evaluated in the Mia PaCa-2 CDX model. **D** IHC analysis of the DR5 expression (upper panels) and H&E staining (lower panels) in the Mia PaCa-2-transplanted CDX tumors. **E**, **F** Tumor growth curve and transplanted tumor weight was evaluated in the PL45 CDX model. **G** IHC analysis of DR5 expression (upper panels) and H&E staining (lower panels) in the PL45-transplanted CDX tumors. IHC and H&E Images were captured at 200× magnification, respectively. Scale bars = 50 µm.

**Fig. 7:**
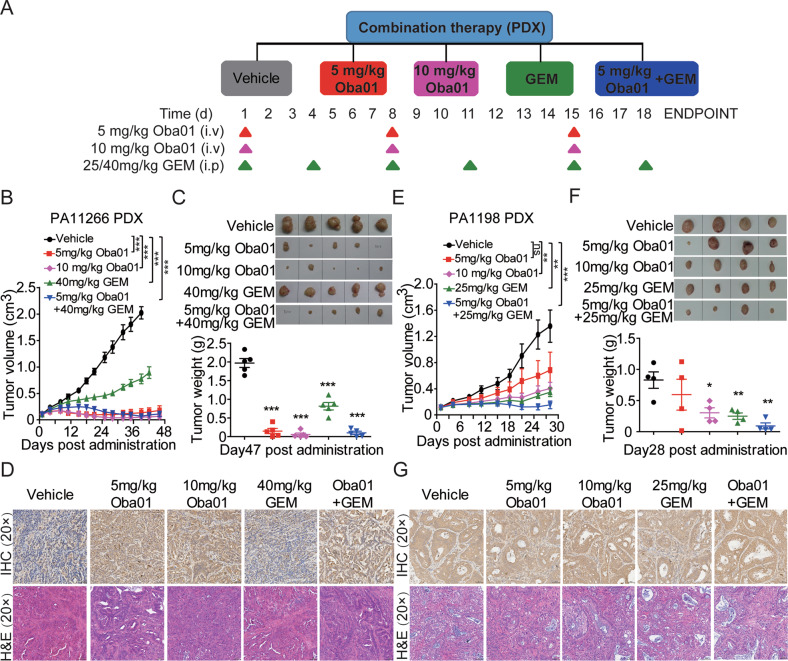
Oba01 and gemcitabine cooperatively inhibited pancreatic cancer PDXs growth. **A** Schematic of the experimental designs. Treatment groups, doses, and schedules are shown. Oba01 (red line, 5 mg/kg), Oba01 (pink line, 10 mg/kg), and vehicle (black line) were administered intravenously and gemcitabine (GEM, green line) was administered intraperitoneally; the combination group is depicted as a blue line. **B**, **C** Tumor growth curve, transplanted tumor weight, and images are displayed in the PA1266 PDX model. **D** IHC analysis of the DR5 expression (upper panels) and H&E staining (lower panels) in the PA1266-transplanted PDX tumors. **E**, **F** Tumor growth curve, transplanted tumor weight, and images evaluated in the PA1198 PDX model. **G** IHC analysis of DR5 expression (upper panels) and H&E staining (lower panels) in PA1198-transplanted PDX tumors. IHC and H&E Images were captured at 200× magnification, respectively. Scale bars = 50 µm.

Next, we evaluated the therapeutic efficacy of Oba01 combined with gemcitabine in CDXs and PDXs. We used a modest therapeutic effective dose of Oba01 and scheduled in these experiments to better identify the synergistic effect of the combination therapy (Figs. 6A, 7A). First, the combination therapy resulted in a significant antitumor effect compared with all other treatment groups in BALB/c nude mice with subcutaneous Mia PaCa-2 xenografts (Fig. 6B, C). CR was observed in five of six animals from days 22–71; only one mouse relapsed at day 55 but continued to show an improved antitumor effect at the end of the experiment on day 71. In contrast, between the two monotherapy groups, treatment with Oba01 significantly delayed tumor progression compared with that with gemcitabine. Similar results were observed in the PL45 CDX model; the combination group showed superior and more significant tumoricidal activity than the monotherapy groups (Fig. 6E, F).

PDX models are generally considered the most relevant for evaluating in vivo antitumor efficacy in preclinical drug development [29, 30]. PA1266 and PA1198 PDAC PDXs were selected to evaluate the antitumor efficacy of Oba01 in vivo. In the PA1266 PDX model, Oba01 induced significantly dose-dependent tumor regression at doses of 5 and 10 mg/kg compared with the vehicle and 40 mg/kg gemcitabine groups (Fig. 7B, C). We observed TGI of 93.33% and 97.46% at doses of 5 and 10 mg/kg, respectively. CR on day 46 was observed in one of five animals in the 5 mg/kg Oba01 dose group. Interestingly, from day 33 to the end of the experiment, the group undergoing combinational therapy showed synergistic inhibitory effects and demonstrated more antitumor activity than the 5 mg/kg Oba01 or 40 mg/kg gemcitabine groups, whereas the therapeutic effect of the combination group was slightly worse than that of the 10 mg/kg dose group. In the other PA1198 PDX model (Fig. 7E, F), a dose-dependent and substantial antitumor activity were observed in the groups undergoing Oba01 treatment compared with the vehicle group, and TGI of 54.22 and 76.69% were observed at doses of 5 and 10 mg/kg, respectively. Notably, the combination of 5 mg/kg Oba01 and 25 mg/kg gemcitabine had significantly higher antitumor activity than other monotherapy groups.

Furthermore, all CDX and PDX tumors were collected and prepared for IHC and H&E assays. DR5 was expressed in all CDX and PDX tumors, indicating that Oba01 antitumor activity was DR5-dependent (Figs. 6D, G, 7D, G). This combination also appeared to be well-tolerated in these CDX and PDX models because no significant body weight changes were observed in these animals (Supplementary Figs. 10,12). In addition, histopathological images of mouse organ dissections showed no toxic adverse effects on the heart, liver, spleen, lung, and kidney (Supplementary Figs. 11,13).

Therefore, these results revealed that gemcitabine could sensitize Oba01-mediated tumoricidal activity in vivo, providing an initial rationale for subsequent clinical development of a combination of gemcitabine and Oba01 as a novel therapy for PC.

## Discussion

DR5 has been currently recognized as a novel target for cancer therapy in solid tumors and hematologic malignancies [20, 31, 32]. We showed that DR5 was expressed in human PC cell lines, CDXs, and PDXs as a biomarker in response to Oba01, which is an ADC. Most PC cells express DR5; therefore, the efficacy of oba01 in vitro was systematically evaluated. In a panel of PC cell lines, Oba01 specifically and dose-dependently inhibited DR5-positive PC cells but not DR5-negative cells. In the DR5 knockout and overexpression assay, we found that DR5 expression is required for Oba01-induced cell death, further indicating that the downregulation or deletion of DR5 may be an important resistance mechanism and that increased DR5 expression may enhance Oba01sensitivity.

IHC analyses of TMA from PADC patient specimens revealed DR5 expression in 50.3% (93/185) of the samples. Moreover, DR5 was highly expressed in all CDX and PDX tumors. We observed different DR5 staining localizations in PADC cells. As observed, DR5 was predominantly localized on the cell membrane and partially retained within the cytoplasm of PDAC tumor cells. Most TMA PC samples from patients showed low DR5 expression. Interestingly, we failed to observe a correlation between DR5 expression and Oba01 efficacy. However, Oba01 showed a superior antitumor effect in the PA1126 model despite the fact that this sample showed low DR5 expression on IHC analyses. Therefore, the overall level of DR5 expression measured using IHC analyses should not be the only selection criteria for patient stratification. Plasma membrane expression may be a more reliable biomarker; however, this should be further explored and validated, and it is practically difficult to perform quantitative expression using surgery and biopsy samples. Furthermore, DR5 expression may require a certain threshold to respond to Oba01, and the in vivo microenvironment may also affect the delivery efficiency and efficacy of Oba01. Therefore, more experiments may be required to define the biomarker selection criteria and determine the lower threshold of DR5 expression required to induce Oba01 antitumor activity.

Combination therapy, in which certain drugs with different mechanisms are coadministered, is widely used in biomedical research and clinical applications. For example, when brentuximab vedotin is combined with a JAK1/2 inhibitor, ruxolitinib, it shows enhanced efficacy against Hodgkin lymphoma [33]. Similarly, PARP inhibitors enhance the antitumor activity of sacituzumab govitecan in triple-negative breast cancer [34] and BCL-2/XL inhibitors enhance the cytotoxicity of T-DM1 [35]. We screened several drug combinations and revealed that a combination of Oba01 and gemcitabine showed the most effective synergy. In vivo results confirmed that Oba01 combined with gemcitabine had a significantly better antitumor effect in CDX and PDX models than standalone gemcitabine or Oba01. Moreover, this combined therapy significantly prolonged survival in the Mia PaCa-2 and PA1266 xenograft models. The optimization of the strategy and efficacy of combination therapies, especially combinations of ADCs with targeted small molecules or immunotherapies, remains a major research topic in the preclinical development of ADC.

**Fig. 8:**
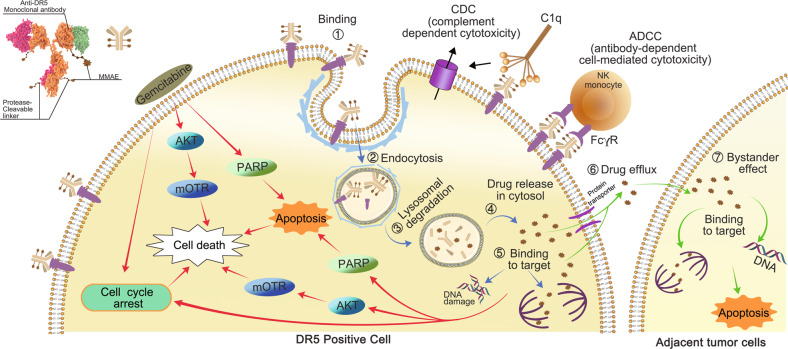
Action mechanism of Oba01. Upon binding to DR5 on the cell surface, Oba01 exerted cytotoxicity through three mechanisms: (1) as indicated by blue arrows, Oba01 is internalized via endocytosis and translocated into lysosome where the MMAE moiety is cleaved off the antibody by proteases, resulting in its release into the cytosol to induce cell apoptosis through DNA damage or microtubule disruption; (2) as indicated by green arrows, cytotoxic MMAEs diffused out of the target tumor cells to kill the neighboring tumor cells that may or may not express DR5, a process known as the “bystander effect”; 3) the antibody moiety of Oba01 promotes natural antitumor immune response mechanisms, such as ADCC and CDC for additional cytotoxicity. As indicated by red arrows, the cytotoxicity of Oba01 is synergistically enhanced when combined with gemcitabine, as both drugs can activate PARP-dependent apoptosis and inhibit pro-mitogenic and pro-survival pathways, such as the PI3K/mTOR-signaling cascade. Gemcitabine can also activate cell apoptosis by inducing DNA damage. Therefore, the combined therapy of Oba01 and gemcitabine could significantly augment PC cell death.

ADCs generally execute antitumor activities by binding to corresponding tumor-associated antigens on the cell surface. They are then endocytosed into lysosomes, where they release the conjugated payload, resulting in apoptosis. Some payloads exert a bystander effect [10]. As indicated in Fig. 8, we enunciated the mechanisms underlying the action of Oba01. Once bound to DR5 on the PC cell membrane, Oba01 is effectively internalized into the lysosome and the payload MMAE is rapidly released by lysosomal proteases. ADC endocytosis and translocation processes have previously been studied and explained [36]. The free form of the payload MMAE is a potent microtubule disruptor and significantly induces G2/M-phase arrest and cell death via apoptosis. Moreover, Oba01 can exhibit bystander effects that inhibit low DR5-expressing cancer cells. Furthermore, the tumoricidal activity of Oba01 is also mediated by ADCC and CDC.

Gemcitabine is reportedly involved in promoting apoptosis by increasing mitochondrial membrane permeability in a ROS-dependent fashion [37]. Gemcitabine can also induce ER stress [38, 39]. Here, we showed that gemcitabine can sensitize the in vitro and in vivo efficacies of Oba01 by synergistically enhancing Oba01-induced PARP-dependent apoptosis, suppressing the cell cycle, and suppressing the PI3K/mTOR pathway activation in various PC models (Fig. 8). The additional involvement of other signaling pathways requires further investigation.

In conclusion, our data indicated that Oba01, the first DR5-targeting ADC, promoted tumor cell death and displayed potent antitumor activity in mono and combinational therapies. It will be valuable to evaluate the efficacy of Oba01 in combination with targeted small molecules or immunotherapies in other solid tumors, which will lead to clinical stage studies of Oba01 as a promising biotherapeutic. Targeted combination therapy can overcome the limitations associated with targeting DR5 agonists in solid tumors and hematological malignancies.

## Supplementary information

AJ-checklist

Supplementary materials

## Data Availability

All data were available within the paper and supplementary materials. Further information and reasonable requests for resources and reagents should be directed to and will be fulfilled by the lead contact, Shuyong Zhang. (zsy206@163.com).
