# Supplementary material for: Therapeutic efficacy of a MMAE-based anti-DR5 drug conjugate Oba01 in preclinical models of pancreatic cancer
Source: Cell Death Dis. 2023 Apr 29;14(4):295. doi: 10.1038/s41419-023-05820-1 (PMC10148860; doi:10.1038/s41419-023-05820-1)
Supplement: Supplementary file 2 — Supplementary materials [file 41419_2023_5820_MOESM2_ESM.docx]

**Supplementary** **materials**

Supplementary materials includes:

Supplementary Tables 1-2

Supplementary Figures 1-13

**Supplementary Table 1**. IC50 of Oba01 against pancreatic cancer Cell lines

|  |  | IC_50_（nM） | | |
| --- | --- | --- | --- | --- |
| Cell line | DR5 expression | Oba01 | Zaptuzumab | MMAE |
| Mia PaCa-2 | + | 17.16±0.21 | >1000 | 0.43±0.13 |
| PL45 | + | 4.79±1.43 | >1000 | 0.22±0.07 |
| PANC-1 | + | 44.06±16.67 | >1000 | 0.20±0.19 |
| PATU 8988 | + | 36.02±9.94 | >1000 | 0.89±0.24 |
| Panc 05.04 | + | 59.29±16.75 | >1000 | 0.62±0.24 |
| Panc 10.05 | + | 347.55±150.54 | >1000 | 0.29±0.31 |
| JF-305 | + | 304.25±14.92 | >1000 | 0.28±0.30 |
| BxPC-3 | + | 200.95±90.58 | >1000 | 0.29±0.30 |
| T24 | - | >1000 | >1000 | 0.06±0.02 |
| Calu-1 | - | >1000 | >1000 | 0.06±0.01 |

**Supplementary Table 2**. Combination study result of drugs showed synergistic effect of Oba01 *in vitro*.

| Name | Primary Mechanism | Target | Approved or in Clinic | Synergy  （Mia PaCa-2） | Synergy  （PL45） |
| --- | --- | --- | --- | --- | --- |
| EKB-569 | EGFR Inhibitor | EGFR | Phase Ⅱ | YES | YES |
| Tubastatin A | HDAC6 Inhibitor | HDAC6 | NO | YES | YES |
| Quizartinib | FLT3 Inhibitor | FLT3 | Phase Ⅲ | YES | NO |
| Trametinib | MEK1/2 Inhibitor | MEK1/2 | Approved | YES | YES |
| BYL-719 | PI3Kα Inhibitor | PI3Kα | Phase Ⅱ | YES | NO |
| AZD7762 | Chk1 Inhibitor | Chk1 | PhaseⅠ | YES | YES |
| Dasatinib | Abl/Src/c-Kit Inhibitor | Abl/Src/c-Kit | Approved | YES | YES |
| Sorafenib | Raf-1/B-Raf Inhibitor | Raf-1/B-Raf | Approved | YES | YES |
| Motesanib | Receptor tyrosine kinase Inhibitor | VEGFR/Kit/  PDGFR/Ret | NO | NO | YES |
| OSI-930 | c-Kit Inhibitor | c-Kit | PhaseⅠ | NO | YES |
| Gemcitabine | Nucleic Acid Synthesis Inhibitor | RRM2 | Approved | YES | YES |

Supplementary Figure 1


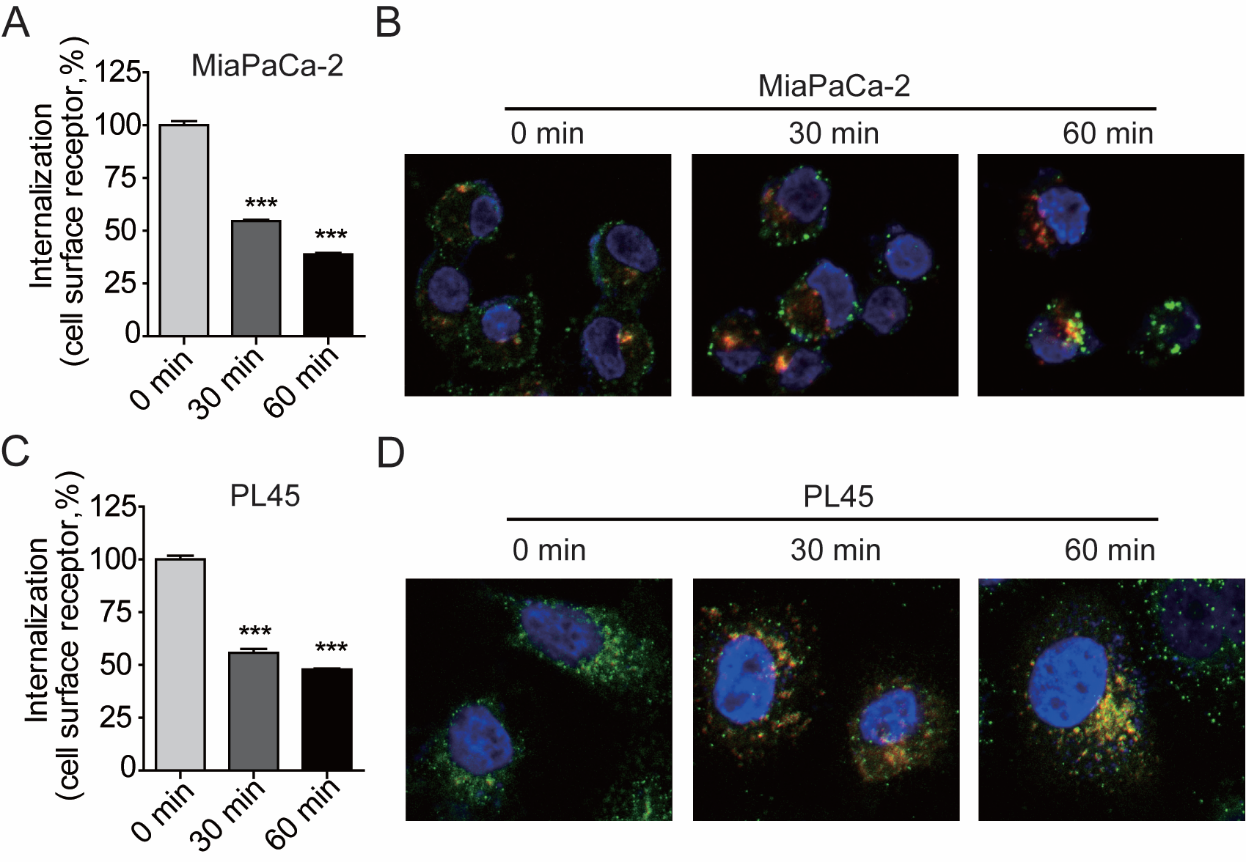


**Supplementary Figure 1. Internalization in pancreatic cancer cell lines.** (A and C) Mia PaCa-2 and PL45 cells were incubated with 2 μg/mL Oba01 for 0, 30 and 60 min at 37 °C, and the cell-surface level of DR5 was determined by measuring surface Oba01 immunoreactivity with flow cytometry. (B and D) The internalization and lysosomal localization of Oba01 in the Mia PaCa-2 and PL45 cells by confocal laser scanning microscope. The cells were treated with 2.0 μg/mL Oba01 at 4 °C for 2h, then incubated for 0, 30 and 60 min in medium at 37°C. The lysosomes were labeled with a LAMP-1 antibody followed by an Alexa Fluor 568-labeled goat anti-rabbit IgG (H+L) antibody. The cell nuclei were stained with Hoechst 33342. Data are means ± SEM.

Supplementary Figure 2


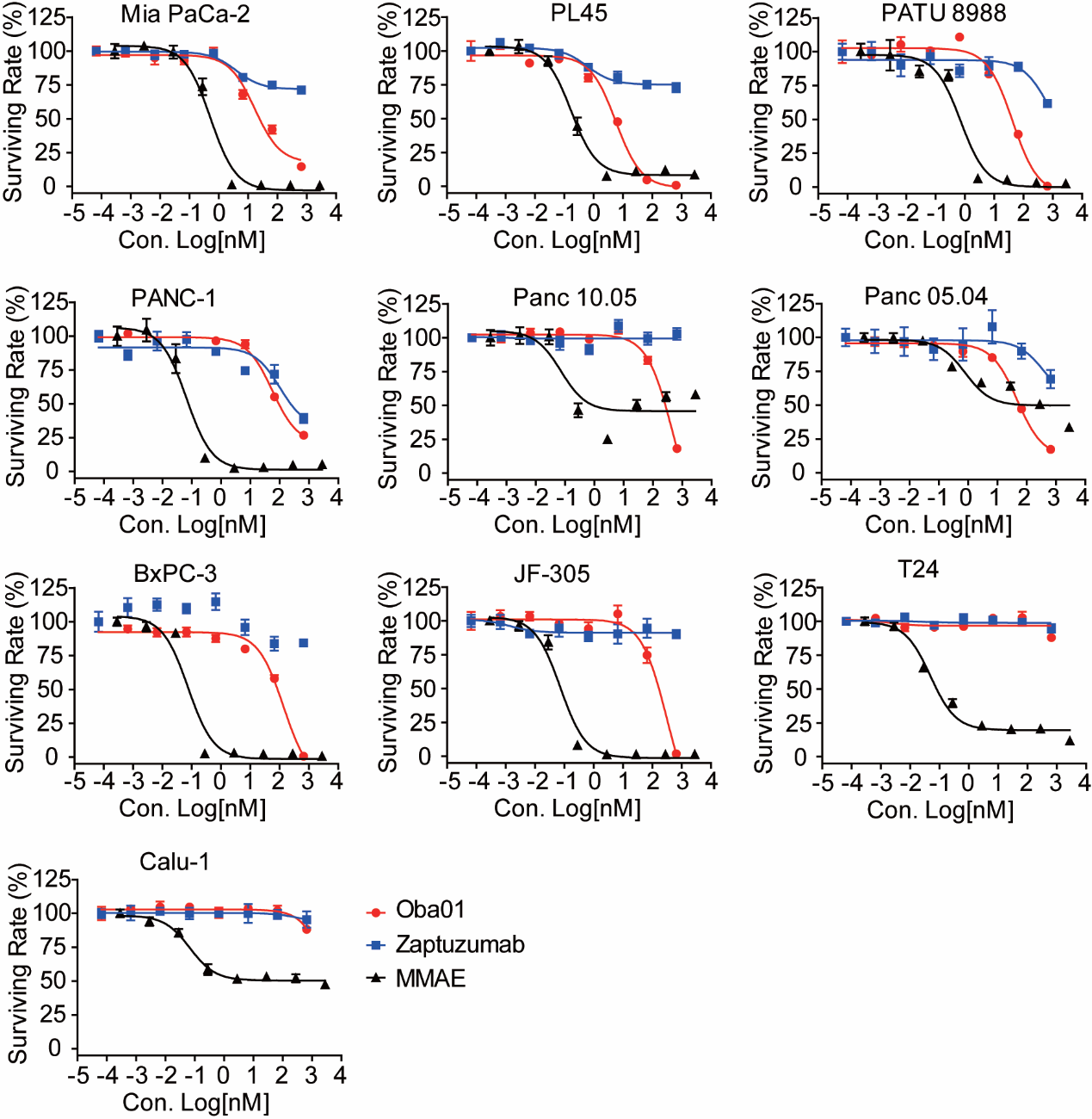


**Supplementary Figure 2.** Cytotoxicity of Oba01, Zaptuzumab and MMAE in a panel of pancreatic cancer cell line, was determined by CellTiter-Glo® Luminescent Cell Viability Assay according to the manufacturer’s instructions. All results are the mean of three independent experiments ± SEM.

Supplementary Figure 3


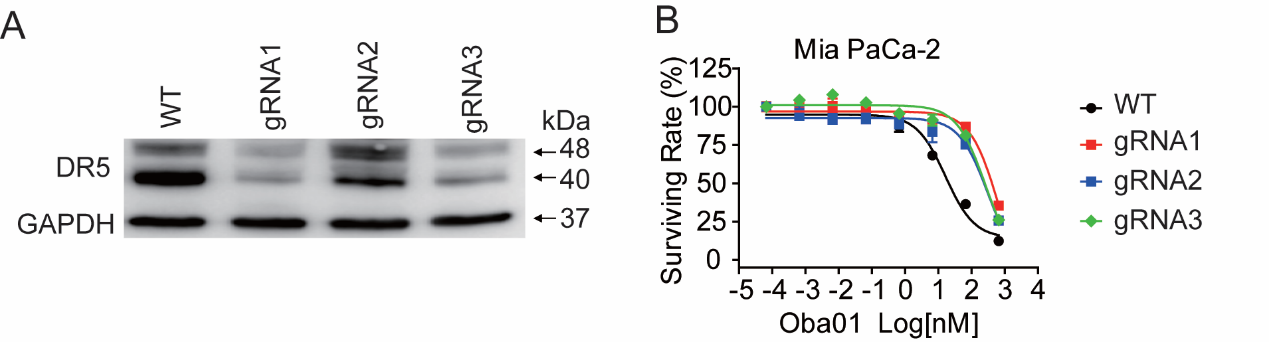


**Supplementary Figure 3.** (A) DR5 protein expression in Mia PaCa-2 control and CRISPRi DR5 knockdown (KD) Mia PaCa-2 cells by western blot. (B) Cytotoxicity assay of Oba01 on Mia PaCa-2 control and DR5 KD cells.

Supplementary Figure 4


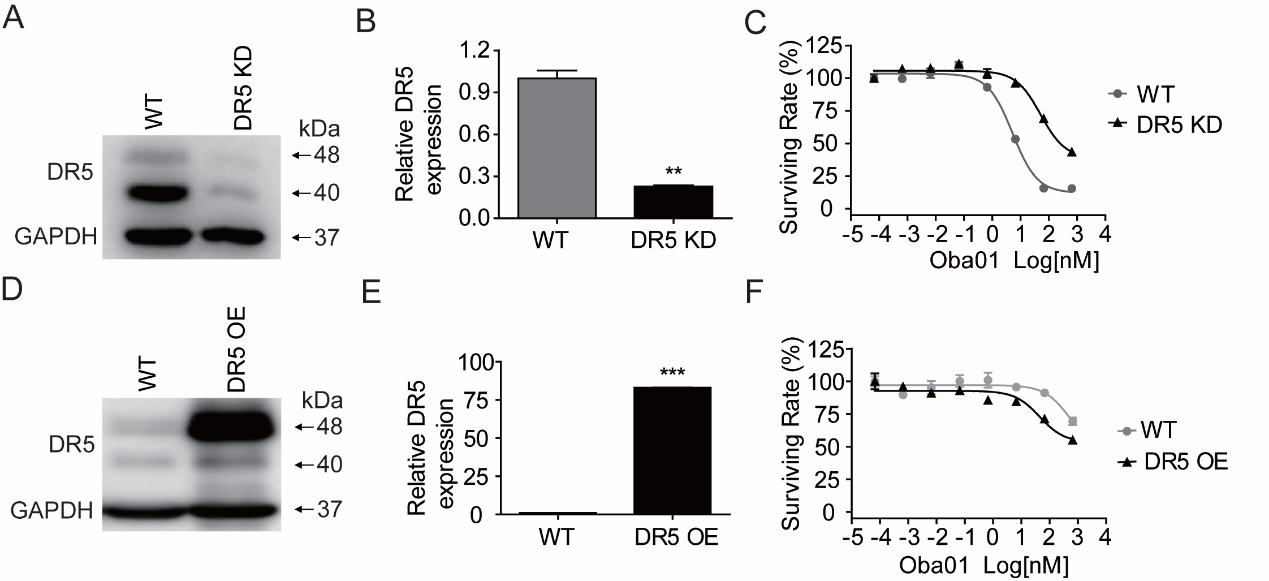


**Supplementary Figure 4. Effects of DR5 knockdown (KD) and overexpression (OE) in PC cells on sensitivity to Oba01.** (A-B) Protein and mRNA expression of DR5 in PL45 control and CRISPRi DR5 KD PL45 cells. (C) Cytotoxicity assay of Oba01 on PL45 control and DR5 KD cells. (D-E) DR5 protein and mRNA expression in Calu-1 control and DR5 OE cells. (F) Cytotoxicity assay of Oba01 on Calu-1 control and DR5 OE cells.

Supplementary Figure 5


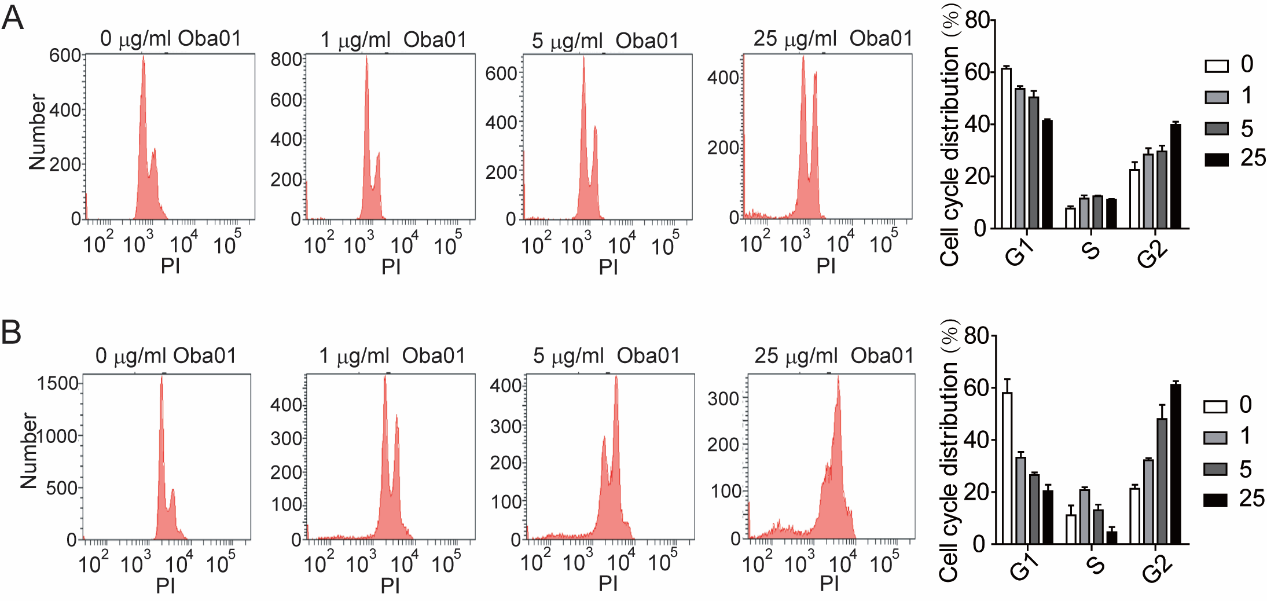


**Supplementary Figure 5.** Effects of Oba01 on cell cycle. The induction of cell cycle analysis in the Mia PaCa-2 (A) and PL45 (B) cells was detected by flow cytometry. The Mia PaCa-2 and PL45 cells were treated with various concentrations of Oba01 for 48 h.

Supplementary Figure 6


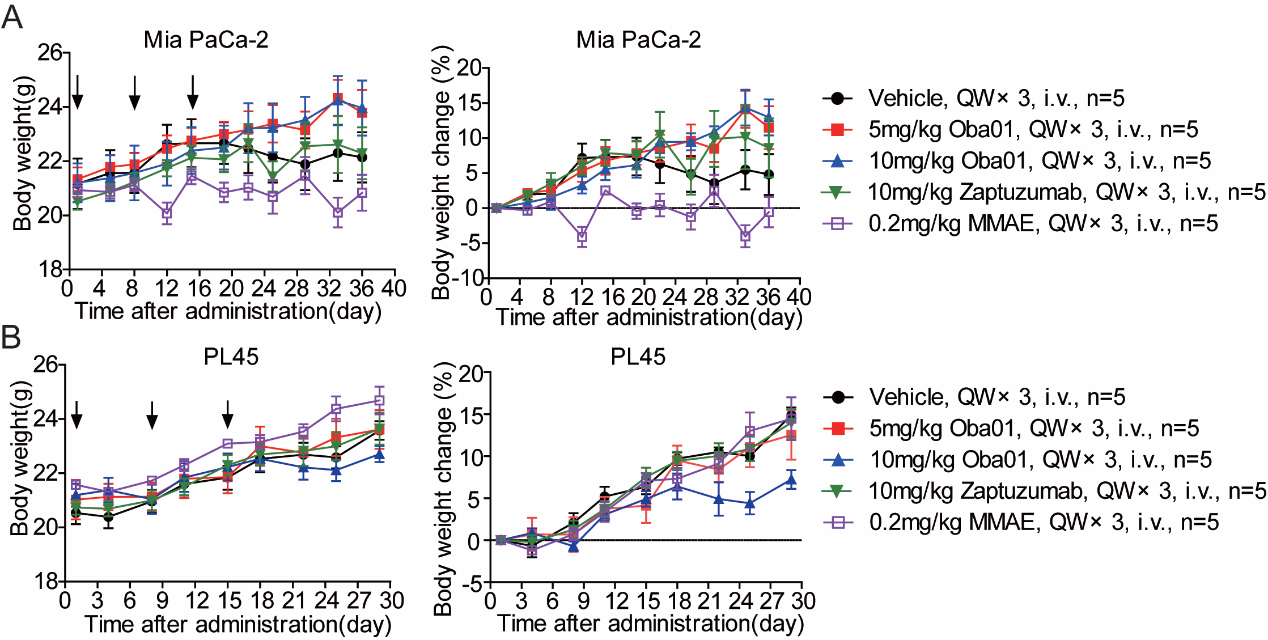


**Supplementary Figure 6.** Mice body weights and the changes of the body weights were assessed twice every week in Mia PaCa-2 (A) and PL45 (B) CDX models, related to Figure 4 as indicated above.

Supplementary Figure 7


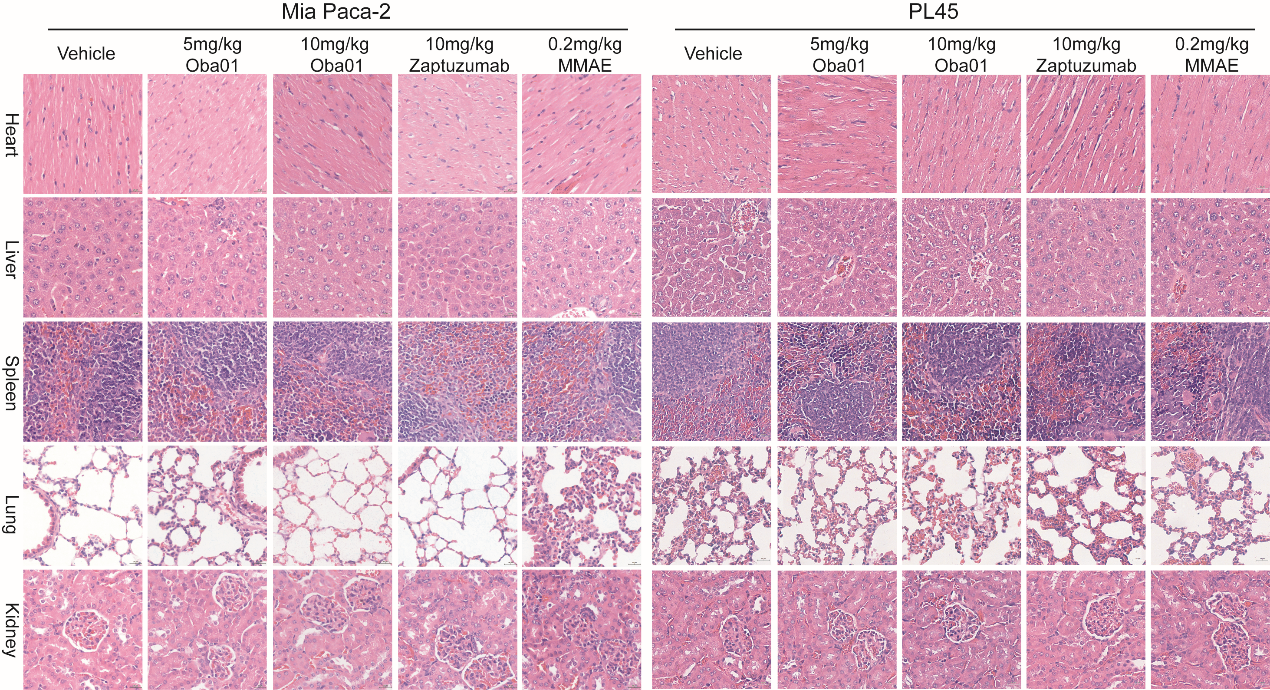


Supplementary **Figure 7**. Treatment of Oba01 had no effects on heart, liver, spleen, lung and kidney in human Mia PaCa-2 (A) and PL45 (B) mouse CDX models. H&E staining assay for the evaluation of pathological changes in these organs of these mouse CDX models, related to Figure 4. Images captured at 400× magnification. Scale bars = 20 µm.

Supplementary Figure 8


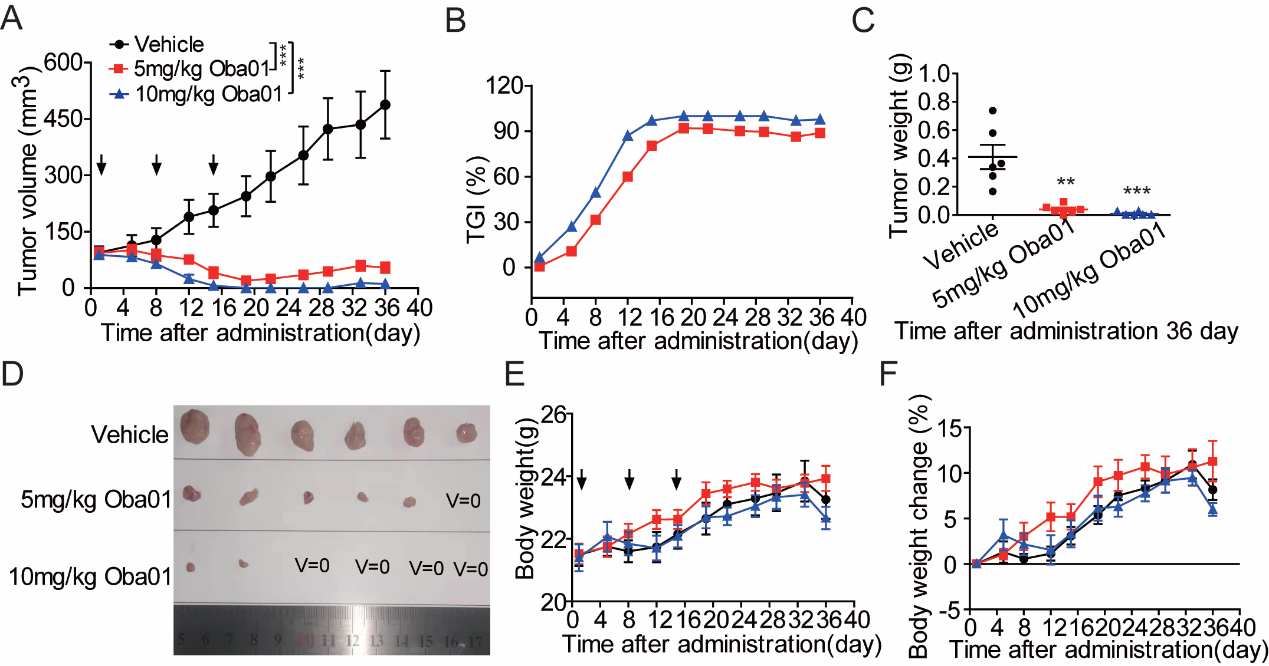


**Supplementary Figure 8.** Antitumor activity of Oba01 in mouse PATU8988 CDX model. BALB/c nude mice were injected s. c. with 1 × 10^6^ PATU8988 cells. Mice bearing xenografts approximately averaged 100-150 mm^3^ were intravenously received saline, 5.0 mg/kg and 10.0 mg/kg of Oba01. Tumor sizes and body weight were measured twice a week. (A-B) Tumor growth curve and inhibition rate of tumor growth (TGI). (C and D) Representative transplanted tumor weights and tumor images were assessed at the end of the experiment. (E and F) Body weights and body weight changes of the CDX mice.

Supplementary Figure 9


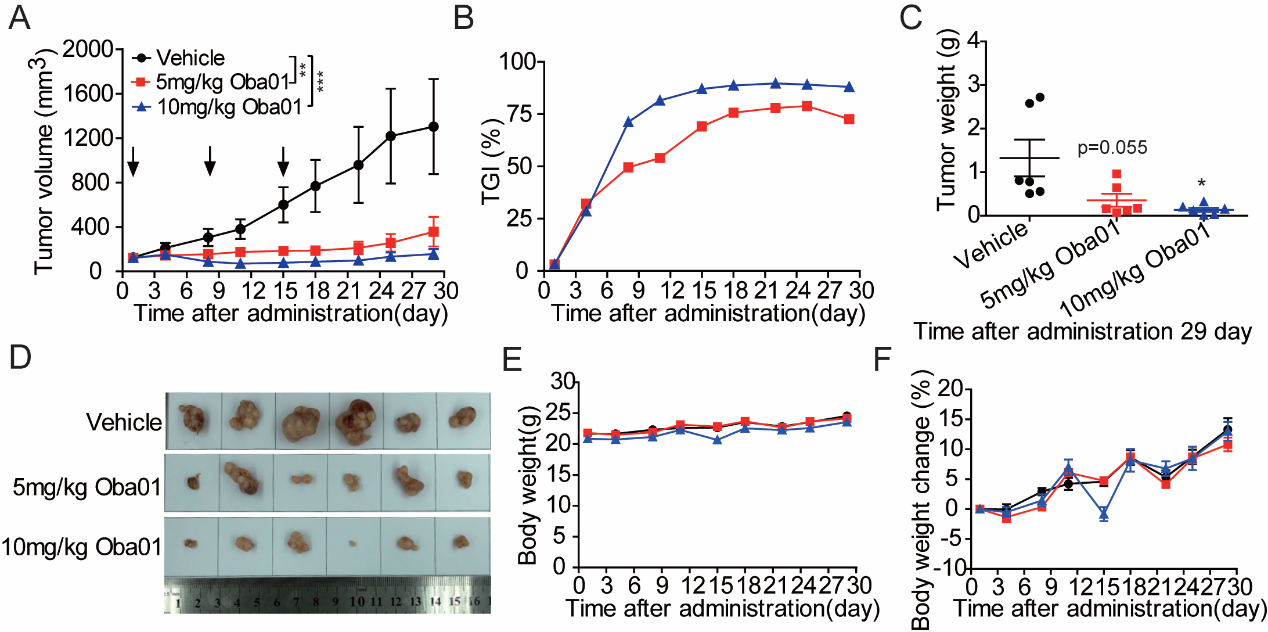


**Supplementary Figure 9. Antitumor activity of Oba01 in mouse JF305 CDX model.** BALB/c nude mice were injected s. c. with 1 × 10^6^ GF305 cells. Mice bearing xenografts approximately averaged 100-150 mm^3^ were intravenously received saline, 5.0 mg/kg and 10.0 mg/kg of Oba01. Tumor sizes and body weight were measured twice a week. (A-B) Tumor growth curve and inhibition rate of tumor growth (TGI). (C and D) Representative transplanted tumor weights and tumor images were assessed at the end of the experiment. (E and F) Body weights and body weight changes of the CDX mice.

Supplementary Figure 10


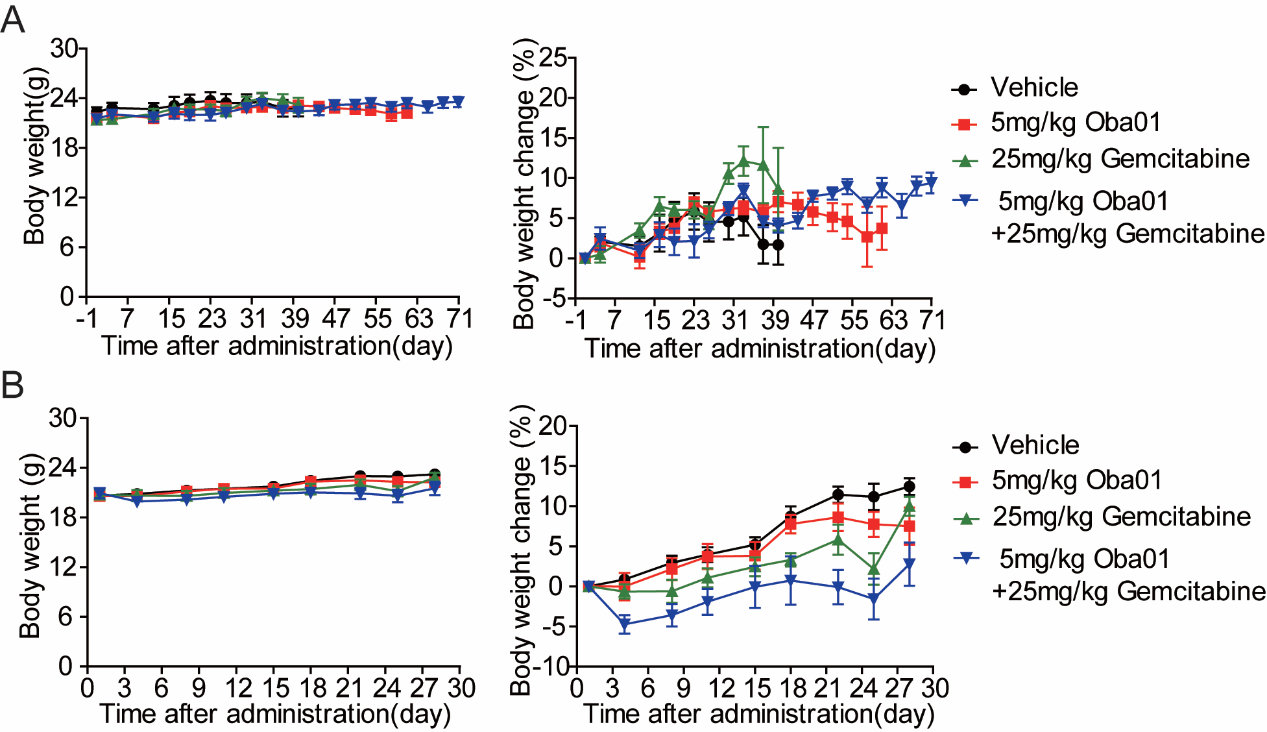


**Supplementary Figure 10.** Mice body weights and the changes of the body weights were assessed twice every week in Mia PaCa-2 (A) and PL45 (B) CDX models, related to Figure 6 as indicated above.

Supplementary Figure 11


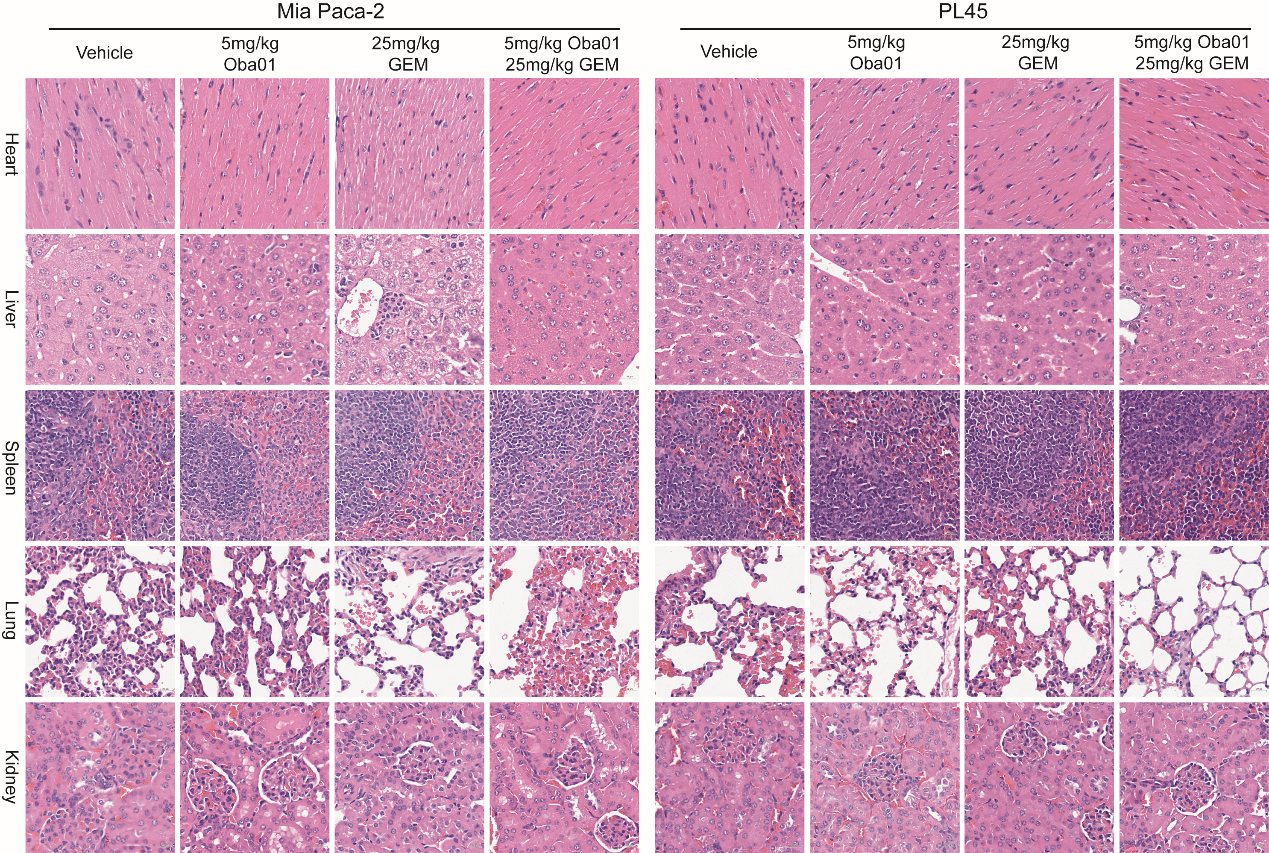


Supplementary **Figure 11**. Combination treatment of Oba01 and gemcitabine (GEM) had no effects on heart, liver, spleen, lung and kidney in human Mia PaCa-2 (A) and PL45 (B) mouse CDX models. H&E staining assay for the evaluation of pathological changes in these organs of these mouse CDX models, related to Figure 6. Images captured at 400× magnification. Scale bars = 20 µm.

Supplementary Figure 12


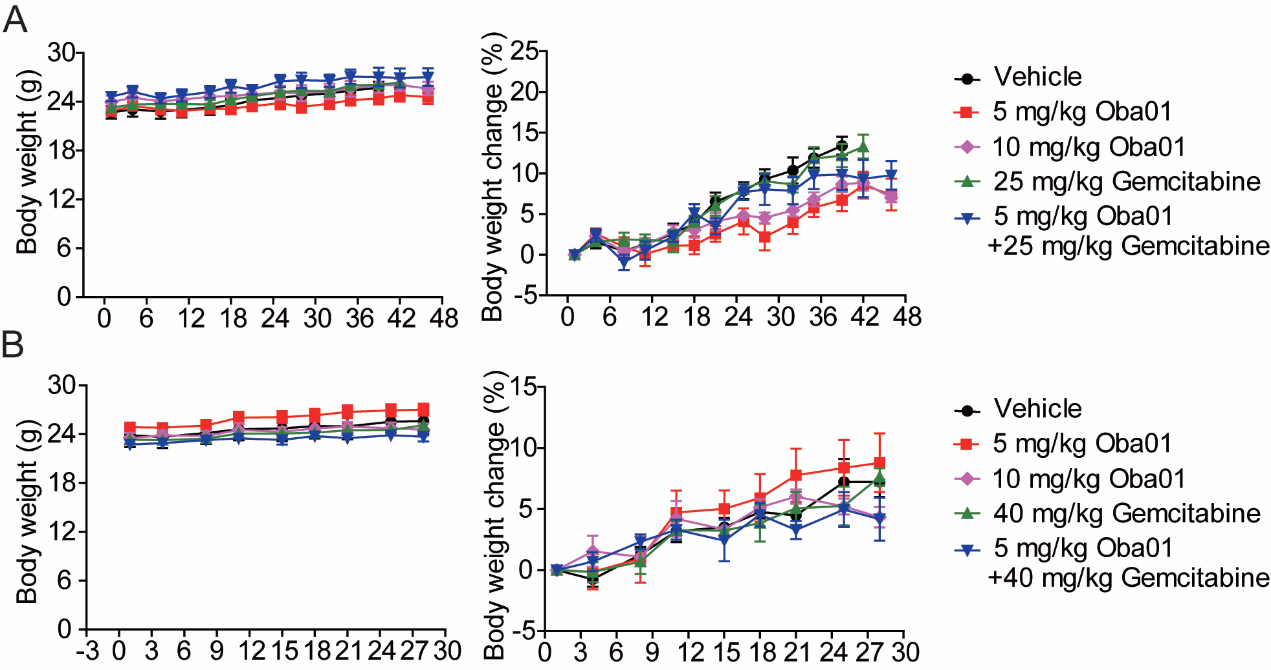


**Supplementary Figure 12.** Mice body weights and the changes of the body weights were assessed twice every week in PA1266 and PA1198 PDX models, related to Figure 7 as indicated above.

Supplementary Figure 13

**
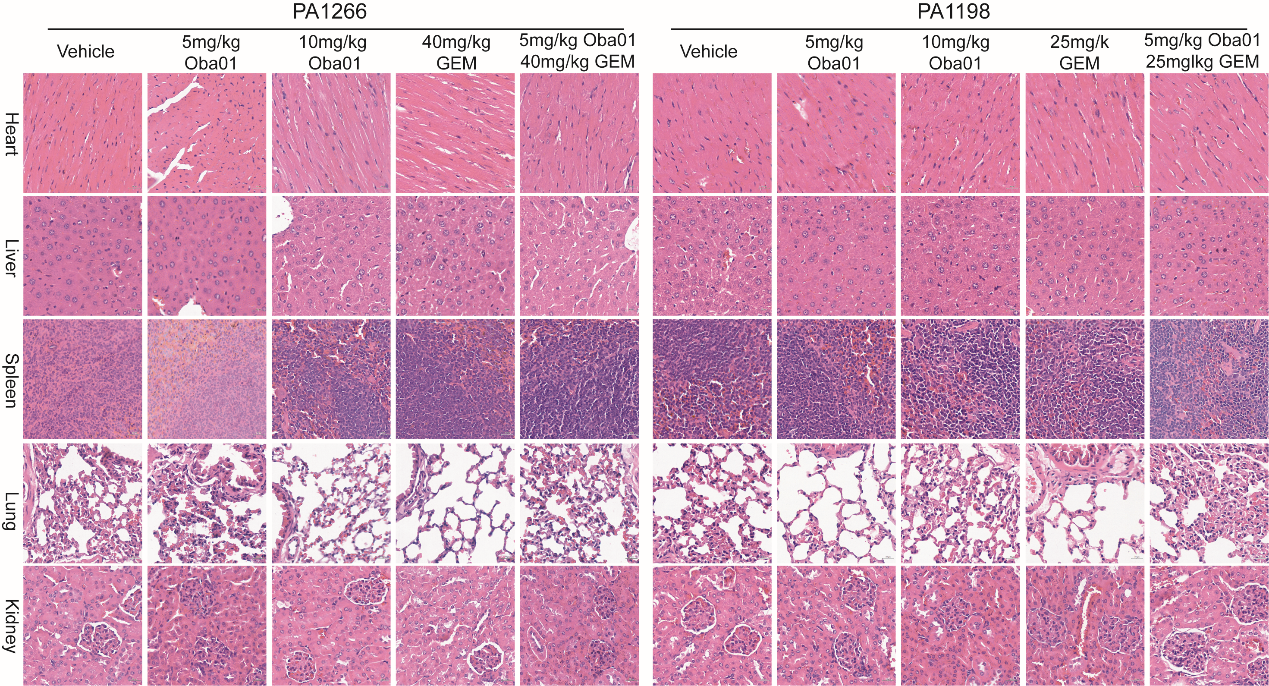
**

Supplementary **Figure 13**. Combination treatment of Oba01 and gemcitabine (GEM) had no effects on heart, liver, spleen, lung and kidney in human PA1266 and PA1198 mouse PDX models. H&E staining assay for the evaluation of pathological changes in these organs of these mouse PDX models, related to Figure 7. Images captured at 400× magnification. Scale bars = 20 µm.
